# Statue to Jenner

**Published:** 1859-09

**Authors:** 


STATUE TO JENNER.—The French nation, by a public subscription, have recently erected a statue in bronze, in front of the Louvre, Paris, to Jenner, the work of M. Eugéne Paul.

